# Metamaterial Reverse Multiple Prediction Method Based on Deep Learning

**DOI:** 10.3390/nano11102672

**Published:** 2021-10-11

**Authors:** Zheyu Hou, Pengyu Zhang, Mengfan Ge, Jie Li, Tingting Tang, Jian Shen, Chaoyang Li

**Affiliations:** 1 School of Information and Communication, Hainan University, Haikou 570228, China; houzheyu@hainanu.edu.cn (Z.H.); pengyuzhang@hainanu.edu.cn (P.Z.); gemengfan@hainanu.edu.cn (M.G.); 2 State Key Laboratory of Marine Resource Utilization in South China Sea, Hainan University, Haikou 570228, China; 3 Key Laboratory of Opto-Electronic Information Technology of Ministry of Education, School of Precision Instrument and Opto-Electronics Engineering, Tianjin University, Tianjin 300072, China; li_jie_d@tju.edu.cn; 4 Information Materials and Device Applications Key Laboratory of Sichuan Provincial Universities, Chengdu University of Information Technology, Chengdu 610225, China; skottt@163.com

**Keywords:** metamaterials, deep learning, reverse design, multiple design

## Abstract

Metamaterials and their related research have had a profound impact on many fields, including optics, but designing metamaterial structures on demand is still a challenging task. In recent years, deep learning has been widely used to guide the design of metamaterials, and has achieved outstanding performance. In this work, a metamaterial structure reverse multiple prediction method based on semisupervised learning was proposed, named the partially Conditional Generative Adversarial Network (pCGAN). It could reversely predict multiple sets of metamaterial structures that can meet the needs by inputting the required target spectrum. This model could reach a mean average error (MAE) of 0.03 and showed good generality. Compared with the previous metamaterial design methods, this method could realize reverse design and multiple design at the same time, which opens up a new method for the design of new metamaterials.

## 1. Introduction

As special human-made materials, metamaterials have attracted the attention of a large number of experts and scholars since they were proposed [1,2]. They allow the design of specific microstructures to change the transmission characteristics of waves to achieve special purposes, including perfect absorption [3,4], hyperbolic materials [5], circular polarization imaging [6,7], and hyperlenses [8]. In addition, based on the chiral phenomenon found in metamaterials, multiple functions have been realized [9,10,11]. The terahertz wave is in the transitional region between electronics and photonics research, and has many unique properties, such as low photon energy and strong penetrability. The emergence of metamaterials makes up for the lack of electromagnetic materials in the terahertz frequency band, and provides an effective way to realize functional devices in the terahertz frequency band. It is also a hot research topic in the field of metamaterials [12,13,14].

Although research on metamaterials is in full swing, designing metamaterial structures on demand is still a challenging task. Researchers must spend a lot of time and computing resources on calculating the effect that a metamaterial with a specific structure can achieve, and then reoptimize the structure of the metamaterial. Even highly experienced researchers cannot easily design a metamaterial structure on demand through traditional methods in a short time.

As an interdisciplinary field covering life sciences, mathematics, psychology, and many other subjects, deep learning has attracted much attention since it was proposed [15]. It allows a model to bypass the basic theory and obtain useful parts from a large amount of data to construct the mapping relationship between input and output. Presently, it has made great achievements in many computer-related fields, including image classification [16], natural language processing [17], and feature recognition [18]. At the same time, there have been many successes in other non-computer-related fields, including many basic disciplines such as chemistry [19], physics [20], and biology [21].

After applying some simple machine-learning algorithms such as a genetic algorithm [22], linear regression [23], and a Bayesian algorithm [24] to the design of metamaterials, deep learning has gradually been applied in this field [25,26,27]. Some researchers have achieved a reverse design for different structures by using different deep-learning networks. However, even within the same structure, there may not be only one set of structural parameters that can achieve the target effect. Therefore, by introducing generative models, including the Variational Autoencoder (VAE), other researchers can calculate other structures that can achieve the same purpose through the designed structure [28,29,30]. However, the method of reverse multistructure design for a certain target effect may be more practical.

In this article, we innovatively proposed a pCGAN based on the idea of the Conditional Generative Adversarial Network (CGAN) [31]. Compared with the method of reverse design using CGAN alone, we obtained a faster training speed, used fewer network parameters, and realized higher accuracy of reverse prediction. The results showed that the use of this model could achieve the purpose of the reverse multistructure design of metamaterials, and the final trained model could reach an MAE of 0.028 (12% lower than for CGANs), and the model showed good robustness and generalization. By using the trained model to guide the design of metamaterials, the design cycle of metamaterials can be effectively shortened. In addition, this model also has high scalability. By only replacing the dataset with the dataset of another structure, the reverse on-demand design of different structures and different target curves can be realized.

## 2. Materials and Methods

### 2.1. COMSOL Simulation Model

We built a simulation model to verify that our network could realize the multiple reverse design of the metamaterial structure. We choose high-resistance silicon (ρ > 5000 Ω∙m) commonly used in the terahertz region as the material to establish a square resonant ring model with four gaps, and observed its effect on the incident light electromagnetic response.

**Figure 1:**
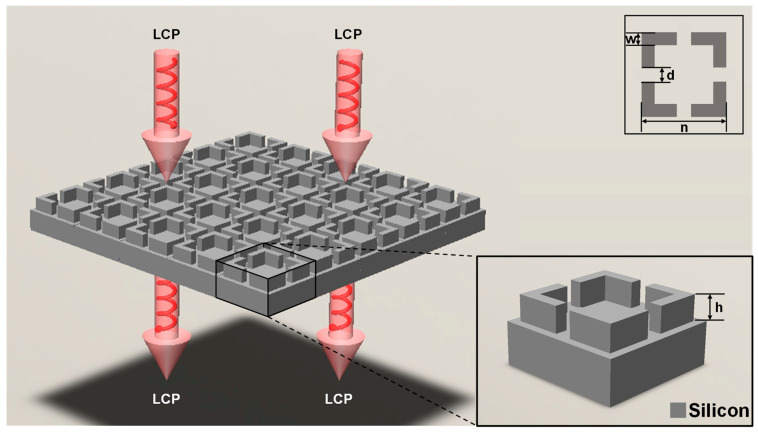
Schematic diagram of the structure. The left-handed circularly polarized (LCP) light was incident perpendicularly to the metasurface to observe its transmittance. The entire super surface was composed of high-resistance silicon.

As shown in Figure 1, the entire metasurface was arranged by meta-atoms with identical structures. Its height h, square outer side length n, square line width w, and gap size d were chosen as structural parameters. The left-handed circularly polarized (LCP) light was incident on this structure perpendicularly, and the frequency-transmittance curves under different structures were obtained by changing the four structural parameters. The size of the super unit substrate was 150 μm × 150 μm, the thickness of the substrate was 50 μm, and the gap position was centered.

The above model was constructed using COMSOL Multiphysics 5.5 [32], and the required dataset by scanning the 4 structural parameters. The scan range of the parameters d and w was (10, 40), the step size was 10, the scan range of the parameters n and h was (30, 110), and the step size was 20. Finally, 400 sets of simulation data were obtained.

### 2.2. The pCGAN Model

When using traditional CGAN for reverse design, neither the generator nor the discriminator can output the spectrum that the structure can produce, which leads to the need to repeatedly use simulation methods to operate the output of the generator during the training process.

On the other hand, the CGAN method is the same as for other Generative Adversarial Networks (GANs). As shown in Formula (1), due to its adversarial loss function construction, the optimal solution requires that the Nash equilibrium be satisfied. The process of seeking the optimal solution requires that the training process between the generator and the discriminator be well synchronized, otherwise the model will be difficult to fit. In addition, when directly using the CGAN method for reverse design, the network training results cannot be intuitively verified through the network, and it still needs to rely on traditional simulation methods, which also makes the training more difficult.


(1) $$\underset{G}{\min}\;\underset{D}{\max}\;V(D,G)=E_{x\sim p_{\mathrm{data}}}(x)[\log(D(x|y)]+E_{z\sim p_{z}(z)}[\log(1-D(G(z|y)|y))]$$

Therefore, we designed a semisupervised-learning deep neural network based on the idea of CGAN, and named it pCGAN. This network included a generator for reverse design and a discriminator for predicting the frequency-transmittance curve that the structure could match. The generator was the main body to realize multiple backward predictions, and the training of the generator relied on an excellent discriminator. The discriminator can be regarded as the loss function of the generator. Therefore, before training the generator, we needed to train the discriminator in a supervised learning method. When the discriminator is trained well, the two networks will be connected, and trained in an unsupervised learning method.

**Figure 2:**
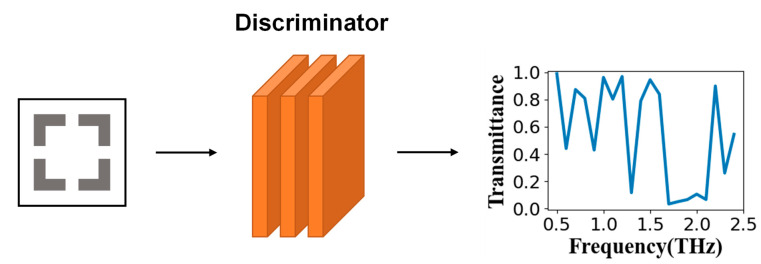
Schematic diagram of the discriminator. The input of the discriminator was the structural parameter, and the output was the corresponding frequency-transmittance curve.

As shown in Figure 2, the discriminator could output the corresponding frequency-transmittance curve by inputting different structural parameters. The well-trained discriminator could obtain the corresponding relationship between the structure parameter and the frequency-transmittance curve. The process of obtaining the frequency-transmittance curve through the trained discriminator took a very short time, and it could be used alone to replace the time-consuming simulation process. Using COMSOL Multiphysics to simulate the single set of structural parameters of the above-mentioned square split resonator ring took about 1 h, while the time for using this network to predict the transmittance curve was only tens of milliseconds. This also allowed the trained discriminator to intuitively reflect the training results of the generator, which had a significant effect on subsequent network optimization and verification.

**Figure 3:**
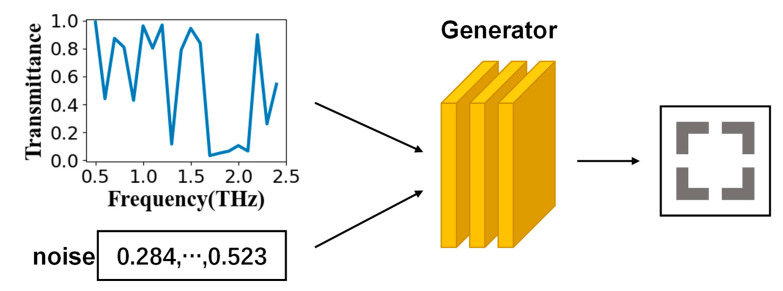
Schematic diagram of the generator. The generator input was random noise and the frequency-transmittance curve, and the output was the structural parameter that could reach the target.

As shown in Figure 3, the generator could output structural parameters that could achieve the required functions by inputting a set of random noise and the required frequency-transmittance curve. The well-trained generator could reversely design different structural parameters that could reach the target curve according to the required frequency-transmittance curve and different random noises. Similarly, the time efficiency of using a trained generator for backward prediction was extremely high.

### 2.3. Neural Network Method

**Figure 4:**
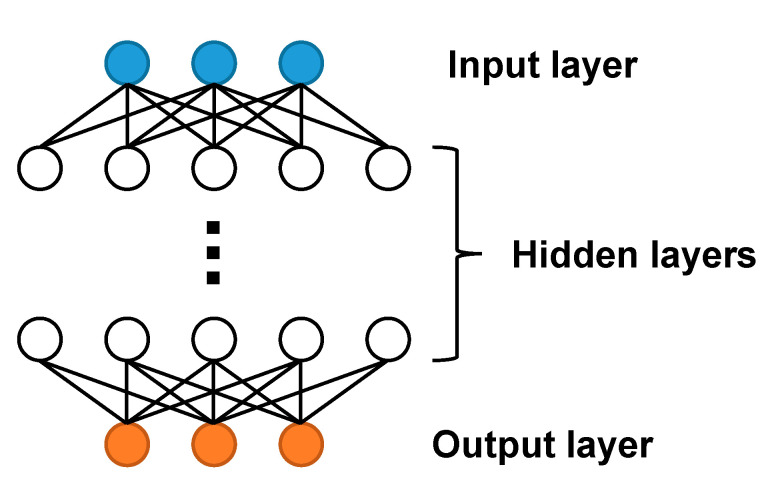
Schematic diagram of a neural network.

As shown in Figure 4, the working principle of the neural network is based on the way the human brain works and learns, and a large number of neural network nodes are constructed to simulate neurons. Each node is connected to adjacent nodes, and the output of the node is adjusted by adjusting the link weight. The output of a single node can be expressed as:

(2) $$y_{j}=\frac{\sum_{i=1}^{n}f(w_{i}x_{i}+b_{i})}{n}$$

where *f* is the activation function, $w_{i}$ is the connection weight between the *i*-th node of the previous layer and this node, $x_{i}$ is the output of the *i*-th node of the previous layer, $b_{j}$ is the bias term of the node, and n is the number of nodes in the previous layer connected to the *j*-th node.

### 2.4. Data Preprocessing

Due to the different dimensions of the input data, it is difficult to maintain the same range of input data. In the process of neural network training, the error caused by the larger input value is usually larger, to avoid the difference between the smaller input value and the larger input value on the network, it is usually necessary to preprocess the input data.

In this work, the transmittance range of different wavelength points in the spectrum data was the same (0 to 1). However, the value ranges of different parameters in the structure parameters were different. It was necessary to perform data preprocessing on the structural parameters. However, after processing them, the structural parameters predicted by the generator also conformed to the rules after preprocessing, so a data preprocessing method that was convenient for restoring the data needed to be used. Here, we choose the Min–Max Normalization data preprocessing method to scale all the original input data $x_{0}$ to the range (0, 1):

(3) $$x=\frac{x_{0}-\min x_{0}}{\max x_{0}-\min x_{0}}$$

This operation not only could avoid the impact on network training caused by different dimensions, but it also helped us to restore the reverse prediction data. The restoration formula is:

(4) $$x_{0}=x(\max x_{0}-\min x_{0})+\min x_{0}$$

### 2.5. Activation Function

To meet the high nonlinearity of the reverse design problem, the Exponential Linear Units (ELU) function that combines the advantages of the Sigmoid and Rectified Linear Unit (ReLU) functions was used as the activation function [33]. The output of the ELU function can be expressed as:

(5) $$f(x)=\{\begin{array}{c} x,\\ \alpha (e^{x}-1), \end{array}\begin{array}{c} x\geq 0\\ x<0 \end{array}$$

where x is the original input, and the parameter *α* ranges from 0 to 1.

**Figure 5:**
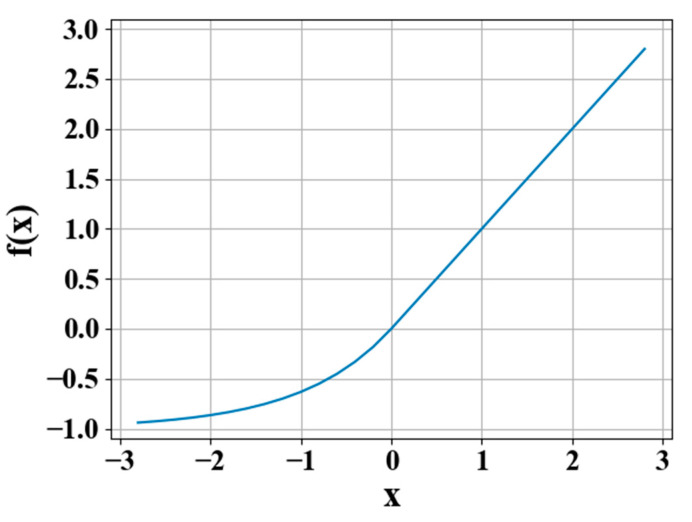
The ELU function curve.

As shown in Figure 5, when x<0, it had better soft saturation, which could make the network more robust to the input; and when x≥0, the gradient was always 1, which was beneficial in alleviating the gradient disappearance of the neural network and making the network easier to converge.

### 2.6. Overfitting Solution

Using a small dataset to train the network will often cause the network to perform worse on data outside the dataset due to the occurrence of overfitting. In order to improve the generalization of the network, L2 regularization was used here to process the weight w, which could make the network learn more features. The regularized output can be expressed as:

(6) $$L=L_{0}+\frac{\lambda }{2n}\sum w^{2}$$

where $L_{0}$ represents the original loss function, and the regularization term $\frac{\lambda }{2n}\sum w^{2}$ is added on this basis, where λ represents the regularization coefficient, n represents the data throughput, and w is the weight. After the regularization term is added, the value of the weight w tends to decrease, and the appearance of excessively large values can be avoided, so it is also called weight decay. L2 regularization can reduce the weight to avoid large slope in the fitted curve, thereby effectively alleviating the overfitting phenomenon of the network and helping to converge.

## 3. Results

### Training of pCGAN

Firstly, we conducted supervised training on the above-mentioned discriminator, and the training set was the simulation data obtained through the COMSOL Multiphysics simulation software. After normalizing the dataset, the order was shuffled, and 10% of it was extracted as the validation set, and the remaining 90% was used as the training set.

**Figure 6:**
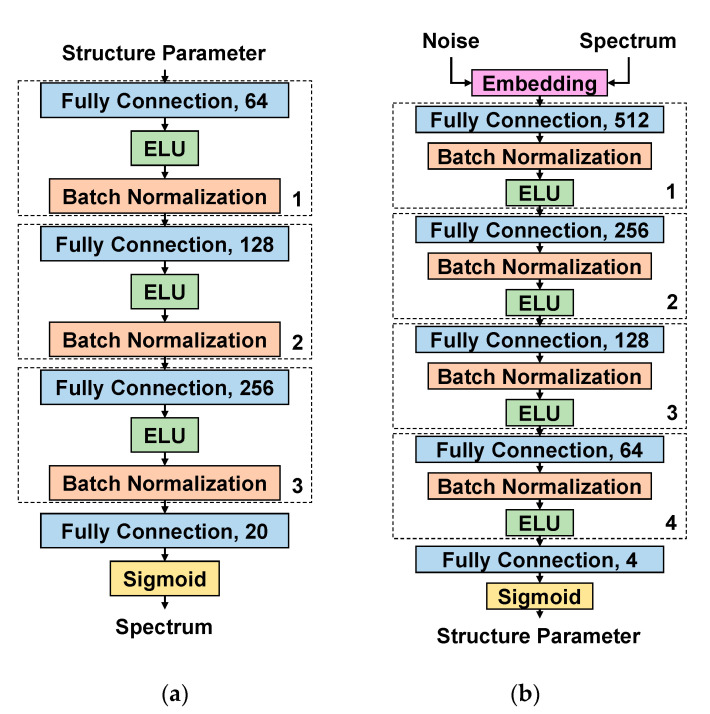
The pCGAN structure parameters. The figure in (**a**) shows the discriminator network structure, and the figure in (**b**) shows the generator network structure.

In the case of reaching the lowest error, we chose the network structure with the least training time. As shown in Figure 6a, the discriminator was composed of one input layer, one output layer, and three hidden layers. The number of nodes in each layer increased with the depth of the network. The hidden layer and the output layer used ELU and Sigmoid as the activation functions, and the L2 regularization method was used to process the weights of each layer to eliminate overfitting. The number of network parameters was 45,524, and the average training time was 1.2 s/epoch.

**Figure 7:**
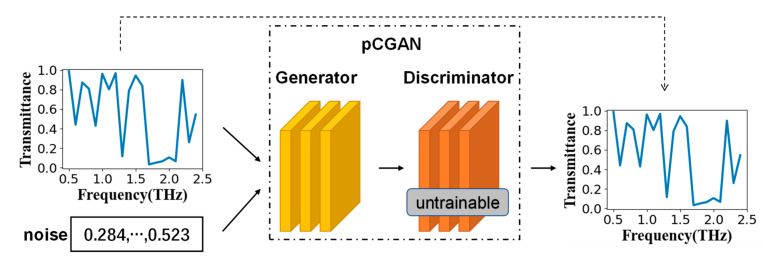
The pCGAN training method. The generator and the discriminator were connected in order, and the discriminator was set to not be trained.

When the discriminator training had been completed, we combined the generator and discriminator as shown in Figure 7, and set the discriminator to be untrainable. Then we inputted different random noise and frequency-transmittance curves for unsupervised training. It should be noted that the input frequency-transmittance curve and the output frequency-transmittance curve were the same. The noise used a random array that satisfied the Gaussian distribution; its mean was 0, its variance was 1, and its size was 4.

As shown in Figure 6b, the generator consisted of one input layer, one embedding layer, one output layer, and four hidden layers that decreased with the depth of the network. Similarly, the hidden layer and the output layer used ELU and Sigmoid (for limited the range of output values) as the activation functions. The embedding layer was responsible for embedding the input noise into the target curve, so that the data of the input hidden layer not only retained the characteristics of the target curve, but also carried the characteristics of random noise. Even if the same target curves were inputted, the generator could still generate different structural parameters according to the different noise. The network parameter amount was 187,432, and the average training time was 244 ms/epoch.

It is worth noting that the networks mentioned above were all running on computers equipped with Intel i7-10750H processors and NVIDIA GeForce RTX 2060 graphics cards.

## 4. Discussion

**Figure 8:**
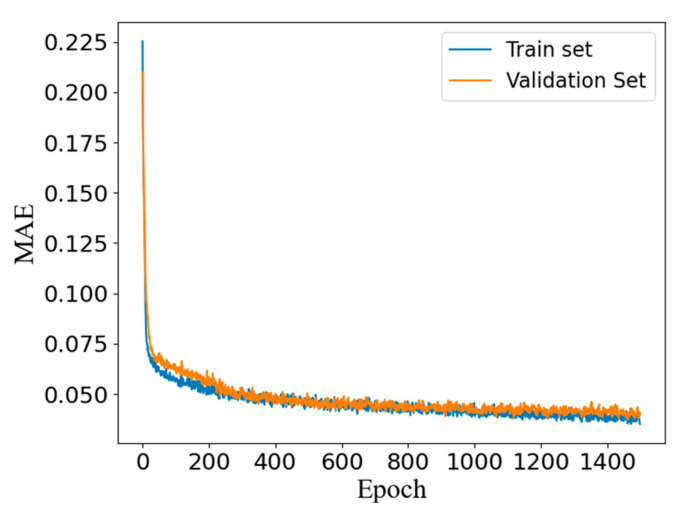
The loss history of the discriminator. The blue line represents the loss of the network on the training set, and the orange line represents the loss of the network on the validation set.

After the discriminator had been trained, there was an obvious overfitting phenomenon, which showed that the error on the verification set was far larger than the error on the training set. This reduced the generalization performance of the discriminator, and the discriminator could not be further trained. After the regularization coefficient λ had been set to 1 × 10^−4^, the overfitting phenomenon was effectively alleviated. In the end, the discriminator reached the optimum after 1500 epochs of training, showing close errors on the training set and the validation set (MAE = 0.033), as shown in Figure 8.

**Figure 9:**
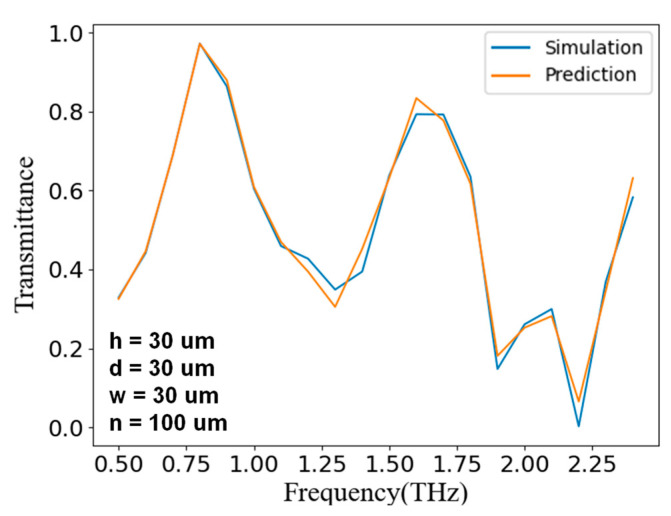
The discriminator training results. The lower left corner shows the input structure parameters, for which the blue line is the corresponding simulation result, and the orange line is the predicted result of the discriminator.

As shown in Figure 9, when we entered the structural parameters shown in the lower left corner to the discriminator, the network output frequency-transmittance curve basically coincided with the simulation result. With this as proof, our discriminator was able to accurately predict the frequency-transmittance curve that the input structural parameters could represent, and the positions and sizes of the peaks and valleys were highly consistent, which achieved the expected purpose. Although there was still a subtle error in the details, it was already within an acceptable range. Using this trained discriminator could effectively guide the training of the generator.

**Figure 10:**
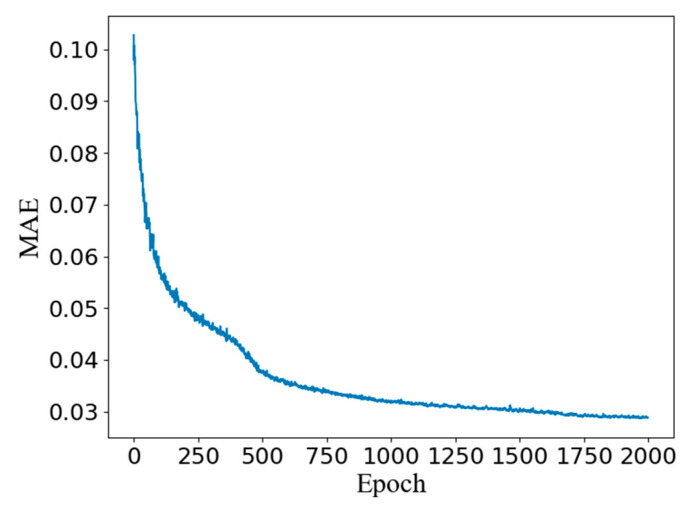
The loss history of the pCGAN.

The training of the generator was non-data-driven unsupervised learning, so there was no need to consider the impact of overfitting, and it reached the best after 2000 epochs of training (MAE = 0.028), as shown in Figure 10.

**Figure 11:**
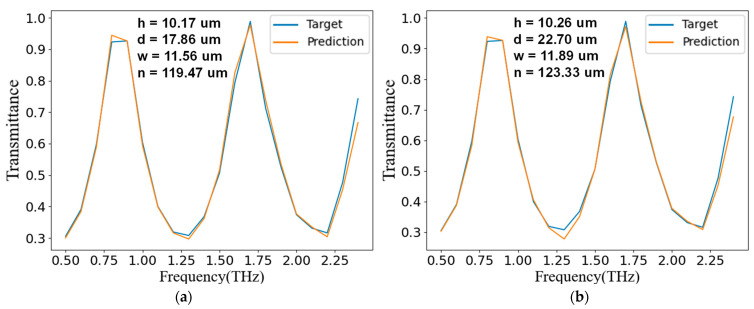
The generator training results. The blue line is the input target frequency-transmittance curve, the upper-center four sets of structural parameters are the output, and the orange line is the frequency-transmittance curve predicted by the discriminator based on the structural parameters output by the generator. The figures in (**a**,**b**) are the running results of inputting the same target curve and different random noises.

Considering that it was difficult to manually draw a set of frequency-transmittance curves that could be achieved by this structure, we selected a set of frequency-reflectance curves in the verification set as the target curve during verification. It is worth noting that this set of curves was not included in the training set, so it was not used when training the network. As shown in Figure 11, when inputting the target curve with two different sets of noise into the generator, the generator could predict two different sets of structural parameters, and both could reach the optical response reflected by the target curve.

**Figure 12:**
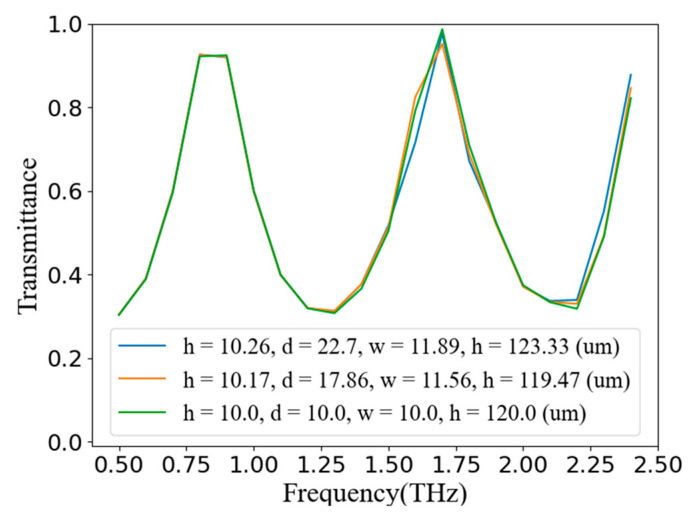
The simulation verification results. All three curves are from the COMSOL simulation results. The blue line is the simulation result produced by the structural parameters outputted by the generator in Figure 11b. The orange line is the simulation result of the structural parameters predicted by the generator in Figure 11a. The green line is the original simulation data and result corresponding to the target curve.

In order to verify the reliability of the generator’s predicted results, we entered the two sets of structural parameters outputted by the generator and the structural parameters that met the target curve from the simulation results into the COMSOL simulation software, and verified them through simulation. As shown in Figure 12, the verification result showed that the effect that our discriminator and generator achieved was remarkable, and achieved the purpose of a one-to-many reverse design.

The above-mentioned reverse multiple design method of pCGAN had good scalability. This method only needs to replace the data of the training discriminator, and can be applied to other different structures, and the target curve is not limited to the frequency-transmittance curve, but can also be applied to the corresponding relationship between phase, incident angle, and absorptance and reflectance. We attempted to verify its performance on different structures, and all the results showed good adaptability. In theory, if a fixed format dataset can be used to characterize different structures and their parameters, this method can be applied to the reverse design of all metamaterial structures. However, because it is not easy to build such a huge dataset, this work is still ongoing.

## 5. Conclusions

In this article, we were inspired by the ideas of CGAN and designed a metamaterial reverse design method based on pCGAN. This method could not only realize the reverse design of the metamaterial structure, but also realized the one-to-many structure design according to the goal. The generator predicted according to the input target curve, and outputted a variety of structures or structural parameters that met the requirements, so that the on-demand design could be solved in this way. Moreover, this method was extremely scalable, and can be widely used in metamaterial design re-search. Experiments have proved that this method is feasible and effective, and the time efficiency is unmatched by traditional design methods.
